# Antiferromagnetic proximity effect in epitaxial CoO/NiO/MgO(001) systems

**DOI:** 10.1038/srep22355

**Published:** 2016-03-02

**Authors:** Q. Li, J. H. Liang, Y. M. Luo, Z. Ding, T. Gu, Z. Hu, C. Y. Hua, H.-J. Lin, T. W. Pi, S. P. Kang, C. Won, Y. Z. Wu

**Affiliations:** 1 Department of Physics, State Key Laboratory of Surface Physics and Collaborative Innovation Center of Advanced Microstructures, Fudan University, Shanghai 200433, People’s Republic of China; 2 Max-Planck-Institut für Chemische Physik fester Stoffe, Nöthnitzer Str. 40, Dresden 01187, Germany; 3 National Synchrotron Radiation Research Center, Hsinchu 30076, Taiwan, Republic of China; 4 Department of Physics, Kyung Hee University, Seoul 130-701, Republic of Korea

## Abstract

Magnetic proximity effect between two magnetic layers is an important focus of research for discovering new physical properties of magnetic systems. Antiferromagnets (AFMs) are fundamental systems with magnetic ordering and promising candidate materials in the emerging field of antiferromagnetic spintronics. However, the magnetic proximity effect between antiferromagnetic bilayers is rarely studied because detecting the spin orientation of AFMs is challenging. Using X-ray linear dichroism and magneto-optical Kerr effect measurements, we investigated antiferromagnetic proximity effects in epitaxial CoO/NiO/MgO(001) systems. We found the antiferromagnetic spin of the NiO underwent a spin reorientation transition from in-plane to out-of-plane with increasing NiO thickness, with the existence of vertical exchange spring spin alignment in thick NiO. More interestingly, the Néel temperature of the CoO layer was greatly enhanced by the adjacent NiO layer, with the extent of the enhancement closely dependent on the spin orientation of NiO layer. This phenomenon was attributed to different exchange coupling strengths at the AFM/AFM interface depending on the relative spin directions. Our results indicate a new route for modifying the spin configuration and ordering temperature of AFMs through the magnetic proximity effect near room temperature, which should further benefit the design of AFM spintronic devices.

Contact between materials with different magnetic orderings can modify their properties near the interface owing to exchange coupling. This phenomenon is usually called magnetic proximity effect. Investigation of the magnetic proximity effect has resulted in the discovery of many new rich physical phenomena^1^, such as the moments induced in heavy non-magnetic metals by proximity to ferromagnets (FMs) reported in a recent magneto-transport study^2^,^3^, and the enhancement of the Curie temperature (Tc) of diluted magnetic semiconductors due to the magnetic proximity effect of FMs^1^,^4^,^5^. The modification of magnetic properties influenced by magnetic proximity effect with FMs has been widely studied. For example, the Tc of FM has been found to be significantly enhanced in FM/FM bilayers^6^,^7^, which could be exploited to optimize magnetic properties in spintronic devices. Another well-known example is the manifestation of a shifted hysteresis loop (i.e., exchange bias)^8^,^9^ combined with enhanced coercivity, stronger magnetic anisotropy^10^,^11^ and spin reorientation transition^12^,^13^ for FMs in ferromagnet/antiferromagnet (AFM) bilayers owing to exchange coupling.

Antiferromagnetic materials, the other class of fundamental magnetic systems, have been widely used in advanced magnetic storage and sensor devices^8^. Recently, spintronic devices based on AFMs have been proposed^14^,^15^,^16^, which are predicted to realize stable high-density memory integration due to their zero moment and other nontrivial properties. However, tuning AFM magnetic properties is considered a great challenge due to its zero moment. Using exchange coupling with neighbor spins in FM/AFM bilayers, the Néel temperature (T_N_) of AFMs can be greatly enhanced^17^,^18^,^19^,^20^, and their spin orientation can be modulated through the formation of a lateral exchange spring spin structure^14^,^15^,^21^,^22^. Nevertheless, the tuning of the magnetic properties of AFMs without FM layer has drawn little attention. So far, there have only been few studies on the modulation of AFM magnetic properties using the AFM magnetic proximity effect in AFM/AFM bilayers. In AFM superlattices comprising two AFM materials with different T_N_, such as [CoO/NiO]_n_^23^,^24^,^25^,^26^ or [FeF_2_/CoF_2_]_n_^27^,^28^, the ordering temperature of the AFM layer with lower T_N_ can be enhanced by the other AFM layer, and the AFM order has a long propagation length of several nanometers. Zhu *et al.* reported that interface coupling can induce AFM spin reorientation transition in the NiO/CoO/MgO(001) system^29^. However, how the spin orientation can be influenced by exchange coupling between AFM spins remains an unsettled question. It is also unclear whether the spin orientation of one AFM layer can affect the T_N_ of the proximate AFM layer.

In this work, in order to detect the AFM properties of top CoO layers, we fabricated a single-crystalline CoO/NiO bilayer on MgO(001) substrate. NiO on MgO(001) substrate has out-of-plane AFM spin orientation owing to tensile strain^30^, whereas CoO on MgO(001) has in-plane AFM spin orientation owing to compressive strain^31^,^32^. Bulk NiO has a T_N_ of ~523 K, and bulk CoO has a lower T_N_ of ~293 K. These distinct spin orientations and T_N_s enable us to study the AFM proximity effect on AFM spin orientation and T_N_ in this system. Using the element-specific X-ray magnetic linear dichroism (XMLD) effect, we demonstrate that the AFM spin orientation of NiO changes from in-plane to out-of-plane with increasing NiO thickness, and the existence of a vertical exchange spring alignment in thick NiO. The spin reorientation transition (SRT) in NiO layer dramatically lowers the T_N_ of the adjacent CoO layer. Further confirmed by systematical magneto-optical Kerr effect (MOKE) measurements of the Fe/CoO/NiO/MgO(001) system, this phenomenon of spin-orientation-dependent T_N_ was attributed to the exchange coupling strength dependent on the spin orientation. The AFM order propagation length was explored by studying the CoO-thickness dependent T_N_. Our results provide clear evidence that the magnetic proximity effect in AFM/AFM bilayers can influence the spin direction and ordering temperature within 2–3 nm of the interface, which is crucial for designing AFM spintronic devices.

## Results

### Antiferromagnetic spin orientation transition in CoO/NiO bilayer

**Figure 1:**
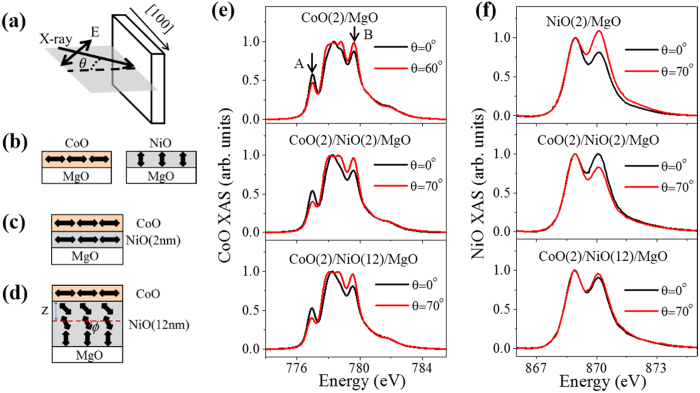
Schematics of XMLD measurement and AFM spin configuration, and obtained XAS spectra. (**a**) Schematic of XMLD measurement geometry. (**b–d**) Schematics of AFM spin configurations with arrows representing the AFM spin alignments. In (**d**), 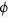 is defined as the spin canting angle between the film surface and spin orientation and z is the depth from the CoO/NiO interface. XAS spectra with 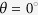 and 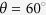 or 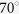 of (**e**) Co^2+^ *L*_*3*_ edge and (**f**) Ni^2+^ *L*_*2*_ edge in NiO(CoO)/MgO(001) and CoO/NiO/MgO(001) samples at 78 K. The unit of the thickness in (**e,f**) is nm. The XMLD measurements of NiO, CoO, and CoO/NiO were performed on different samples.

We applied XMLD measurements to determine the AFM spin orientation in a single-crystalline CoO/NiO bilayer on a MgO(001) surface using the total electron yield (TEY) mode. In XMLD measurements, the obtained X-ray absorption spectrum (XAS) is strongly dependent on the relative orientation of the AFM spin axis and the linear polarization vector of the X-rays used^29^,^30^,^31^,^32^,^33^,^34^,^35^,^36^. Thus, to determine whether the AFM spin was in-plane or out-of-plane, XAS was measured as a function of the incident angle (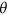), defined as the angle between the propagation direction of the X-rays and the direction normal to the sample surface, as shown in Fig. 1(a). To investigate the magnetic properties of the CoO layer, CoO *L*_*3*_ edge spectra were measured with X-rays at normal incidence (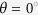) and grazing incidence (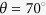 or 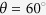). Figure 1 shows the results of XMLD measurements performed at 78 K, which is much lower than the T_N_ of both NiO and CoO films in our samples^13^,^29^,^35^. As shown in Fig. 1(e), the XAS spectra observed for 2 nm CoO proximate to 2 nm and 12 nm NiO were similar to that of CoO/MgO, which exhibits in-plane spin orientation^29^,^32^. Thus, at low temperature, the CoO AFM spin was aligned in-plane on top of both 2 nm and 12 nm NiO. Figure 1(f) shows the typical XAS measured at the Ni^2+^ *L*_*2*_ edge, in which the doublet spectra have been normalized to the intensity of the lower energy peak. The second peak of the XAS obtained from the 2 nm NiO in the CoO/NiO bilayer exhibited a clearly reversed intensity for 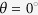 and 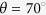 compared with those from the NiO film grown on MgO substrate, which indicates that AFM spin of 2 nm NiO was aligned out of plane on MgO substrate^29^,^30^ [Fig. 1(b)] and in plane in AFM bilayer [Fig. 1(c)]. However, the second peak in the NiO XAS spectrum of the CoO (2 nm)/NiO (12 nm) bilayer showed similar intensity at 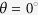 and 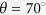. Thus, we can conclude that the AFM spins of the 12 nm NiO were in the canting state with comparable in-plane and out-of-plane components. This result is significantly different from that reported for a reversed growth order in NiO (12 nm)/CoO bilayer on MgO(001) substrate, which showed out-of-plane AFM spin orientation of the NiO^29^. Due to the limited electron escape length in NiO^35^, the observed intensity of the XAS measurements in TEY mode mostly came from a several nanometer thickness of the NiO films. Thus, the XAS from the 12-nm NiO layer shown in Fig. 1(f) reflects the properties of the NiO near the CoO/NiO interface, whereas previous measurements on NiO/CoO bilayers only revealed the information for the NiO away from the NiO/CoO interface^29^.

**Figure 2:**
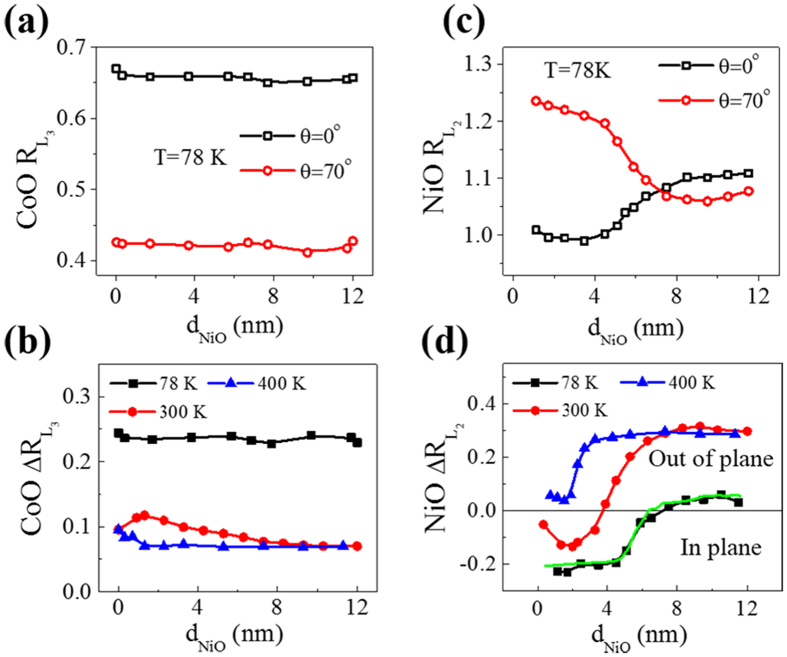
Change of CoO AFM order and NiO spin reorientation transition with increasing NiO thickness. (**a**) CoO *L*_*3*_ ratio and (**c**) NiO *L*_*2*_ ratio with 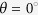 and 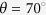 as a function of NiO thickness at 78 K. Difference in (**b**) CoO *L*_*3*_ ratio and (**d**) NiO *L*_*2*_ ratio as a function of NiO thickness at 80 K, 300 K, and 400 K. The green line in (**d**) was calculated based on Monte Carlo simulation results.

The XMLD effect of CoO can be quantified by the *L*_*3*_ ratio 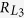, which is defined as the ratio of the intensities of the XAS peaks at 777 eV and 779.6 eV [marked as A and B in Fig. 1(e), respectively]^32^. For NiO, the XMLD effect is quantitatively described by the NiO *L*_*2*_ ratio 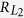, which is defined as the ratio of the intensity of the lower energy peak divided by that of the higher energy peak^30^,^35^. Figure 2(a,c) respectively show the NiO-thickness-dependent CoO *L*_*3*_ ratio 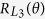 and NiO *L*_*2*_ ratio 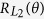 measured at incidence angles of 0^o^ and 70^o^ at 78 K. In Fig. 2(a), the CoO 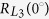 is always larger than 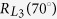 at various NiO thicknesses. Consequently, the difference of the CoO *L*_*3*_ ratio 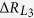

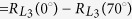
 is positive for all NiO thicknesses in Fig. 2(b). This result indicates that the CoO spin aligns in plane irrespective of the NiO thickness, which may be attributed to the large anisotropy of the CoO layer^37^,^38^. The XMLD of the NiO film showed a more complicated behavior. In Fig. 2(c), 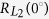 was smaller than 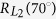 for NiO thicknesses smaller than 4 nm, after which 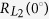 began to increase while 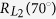 began to decrease, and they crossed over at d_NiO_~7.0 nm. Correspondingly, in Fig. 2(d), the difference of NiO *L*_*2*_ ratio, 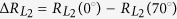, changed from a negative value to a small positive value at 78 K. It has been shown that the NiO *L*_*2*_ ratio 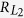 should be smaller for 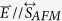 than 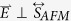 when the NiO spin is aligned along the <100> direction^29^,^30^,^34^. Thus, the data in Fig. 2(c,d) indicate that the NiO spin orientation switched from in-plane to a canting orientation at 78 K. NiO-thickness-dependent experiments were also performed at 300 K and 400 K. As shown in Fig. 2(d), the NiO 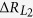 at 300 K changed from negative to positive, indicating that the NiO spin switched from in-plane to out-of-plane. The NiO 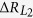 at 400 K showed a rapid increase at d_NiO_~2.0 nm, which was related to the establishment of NiO AFM order^29^.

### NiO-spin-orientation-dependent enhancement of CoO T_N_

**Figure 3:**
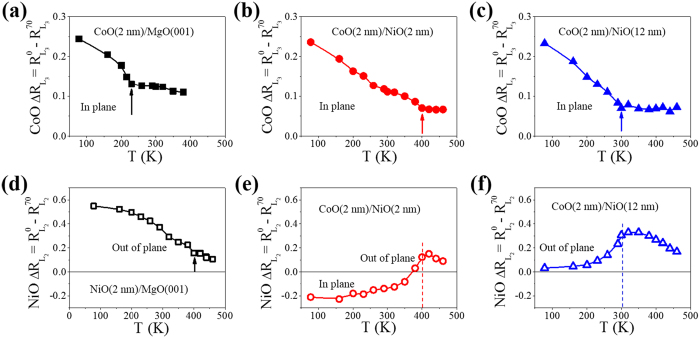
Spin-orientation-dependent Néel temperature by XMLD measurements. Temperature dependence of (**a**–**c**) CoO *L*_*3*_ ratio difference 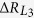 and (**d**–**f**) NiO *L*_*2*_ ratio difference 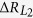 for the systems CoO/MgO(001), NiO/MgO(001), and CoO/NiO/MgO(001) with layer thicknesses indicated on the top of each panel. Arrows indicate the AFM ordering temperature T_N_.

The XMLD effect observed at the Co^2+^ *L*_*3*_ edge and Ni^2+^ *L*_*2*_ edge can generally be attributed to AFM ordering and the crystal-field effect^33^,^39^. While the AFM contribution decreases with increasing temperature and vanishes above T_N_, the contribution of the crystal field only slightly decreases at higher temperature. Thus, the T_N_ of the AFM layer could be identified from the transition point of the CoO 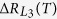 curve and NiO 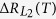 curve^29^. Temperature-dependent XMLD measurements were performed as shown in Fig. 3. In Fig. 3(a–c), the Co^2+^ 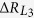 was always positive, indicating in-plane aligned Co^2+^ AFM spins. The different CoO 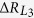 value at high temperature could be attributed to a change of the crystal field influenced by different strain in the CoO layer. The single 2-nm CoO layer exhibited a T_N_ of 230 K [Fig. 3(a)]. When the CoO layer was proximate to 2 nm NiO, its T_N_ was dramatically enhanced to 400 K [Fig. 3(b)], whereas the T_N_ of the CoO layer on top of 12 nm NiO was only enhanced to 300 K [Fig. 3(c)]. This result seems unexpected because the Néel temperature of 12 nm NiO is evidently higher than that of 2 nm NiO owing to the finite-size scaling^13^.

Using the advantages of XMLD with elementary specification, we also measured the temperature-dependent 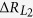 of the NiO layer in CoO/NiO bilayer as shown in Fig. 3(d–f). Our measurements indicated that the 2 nm NiO had a T_N_ of ~400 K, which coincides with the changes in the NiO 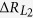 seen in Fig. 2(d). Owing to the temperature-dependent crystal field effect^31^,^33^, the 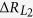 of the 2 nm NiO still decreased above its T_N_. The 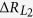 of 2 nm NiO in the AFM bilayer was negative at low temperature and became positive approaching 400 K, which indicates that the NiO spin had in-plane orientation at low temperature, and then switched to out-of-plane at high temperature [Fig. 3(e)]. In Fig. 3(f), the 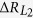
of 12 nm NiO had a small positive value at low temperature, and started to increase when the temperature approached 300 K. This indicates that the NiO spin at the CoO/NiO interface changed from the canting state to an out-of-plane oriented state. Note that the SRT of the NiO layer occurred exactly at the T_N_ of the proximate CoO layer, which could be ascribed to the decreased interfacial coupling strength in the AFM bilayer related to the weakened CoO AFM order. Based on these results, we can conclude that the NiO layer with in-plane aligned spin orientation largely enhanced the T_N_ of the adjacent CoO layer by 170 K whereas the NiO layer with out-of-plane spin orientation only enhanced the T_N_ of the CoO layer by 70 K. It should be pointed out that, as a result of thickness dependent lattice relaxation, the strain of the film grown on thick NiO may be different to that of the film grown on thin NiO. However, the observed different T_N_s are unlikely to be attributable to the different strains of the two systems. High energy electron diffraction (RHEED) patterns of the 2- and 12-nm NiO films showed that the difference in lattice constant was less than 0.6%. However, as reported in ref. 31, while the difference in lattice constant between bulk MnO and Ag can be 8.2%, the T_N_ of CoO film grown on Ag(001) substrate is only 20 K higher than that grown on MnO(001) film.

**Figure 4:**
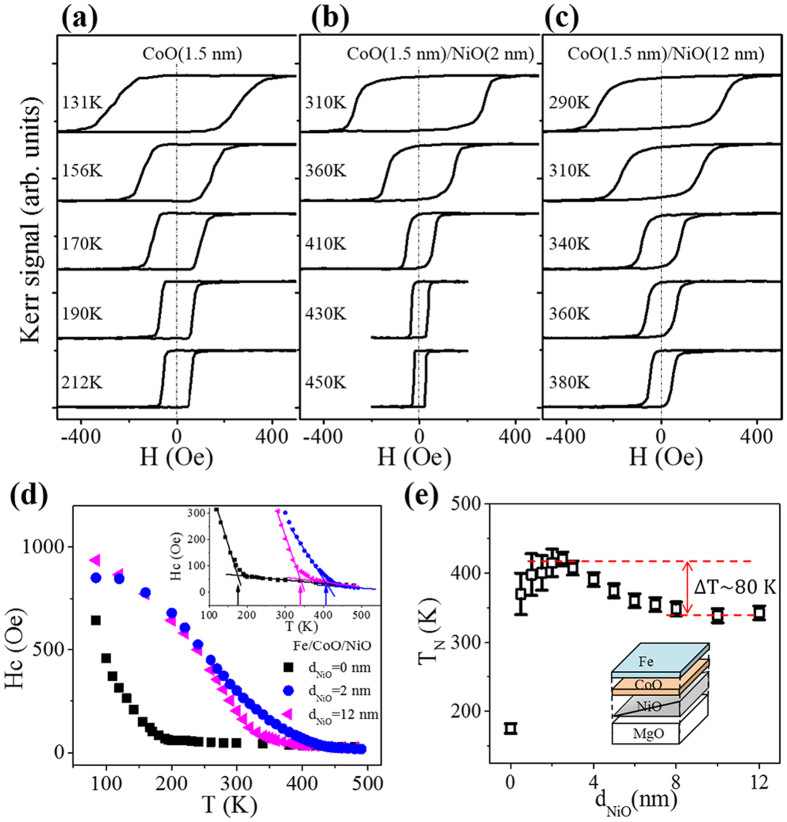
Spin-orientation-dependent Néel temperature by MOKE measurements. Typical hysteresis loops for the systems (**a**) Fe/CoO/MgO(001) and (**b,c**) Fe/CoO/NiO/MgO(001) with indicated layer thicknesses at different temperatures. (**d**) Temperature-dependent Hc of Fe/CoO/NiO/MgO(001) with different NiO thickness. Inset shows an enlargement of the Hc around T_N_. Arrows denote the CoO T_N_ evaluated from the discontinuity of the slopes of the Hc(T) curves. (**e**) CoO T_N_ as a function of NiO thickness for the Fe/CoO/NiO(wedge)/MgO(001) sample. Inset in (**e**) shows the structure of the sample.

In order to systematically investigate how the T_N_ of the CoO film changed with NiO thickness, a sample of Fe (2 nm)/CoO(1.5 nm)/NiO/MgO(001) was prepared with a wedge-shaped NiO layer. It is well known that below the T_N_, the antiferromagnetic spins in a FM/AFM bilayer can dramatically increase the coercivity (Hc) of FM layer through the exchange coupling between the FM layer and AFM layer. Thus, the T_N_ of the CoO layer could be determined from temperature-dependent behavior of its Hc^8^,^20^. Figure 4(a–c) show typical hysteresis loops obtained at different temperatures for Fe/CoO, Fe/CoO/NiO (2 nm), and Fe/CoO/NiO (12 nm), respectively. The samples exhibited easy hysteresis loops with negligible exchange bias field under a sweeping field *H* parallel to the cooling field 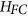 along the CoO<110> directions^11^,^32^. The temperature-dependent Hc is plotted in Fig. 4(d), the inset of which shows the enlarged region around the T_N_ of the CoO layer, which is defined as the discontinuity point of the slope of the Hc(T) curve. From this plot, the T_N_ of the 1.5-nm CoO single layer can be judged to be about 175 K. For CoO on top of 2 nm NiO, the T_N_ was enhanced to 415 K, while that of CoO on top of 12 nm NiO was enhanced to 343 K.

Figure 4(e) shows the results of the systematical measurement of CoO T_N_ as a function of NiO thickness. The T_N_ of the 1.5-nm CoO layer first increased to ~400 K with NiO thickness, began to decrease for NiO thicker than 4 nm, and finally saturated to ~340 K for NiO thicker than 8 nm. The T_N_ of CoO decreased by ~80 K on thicker NiO, which is consistent with the XMLD results. Recalling the fact that the SRT of NiO occurred at a thickness range of 4–8 nm at temperatures of 300–400 K in Fig. 2(d), the results here further confirm that the T_N_ of the CoO layer was closely related to the spin orientation of the proximate NiO layer. Therefore, in Fig. 2(b), for the CoO layer on top of thick NiO film, the 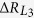 for CoO at 300 K is similar to that observed at 400 K, indicating the paramagnetic state of CoO at 300 K. However, for CoO layers proximate to NiO layers thinner than 6 nm, we found a small difference in the CoO 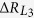 measured at 300 K and 400 K, which indicates that the AFM order of the CoO layer on top of thin NiO film persisted above 300 K.

### Propagation of CoO AFM order in CoO/NiO bilayer

**Figure 5:**
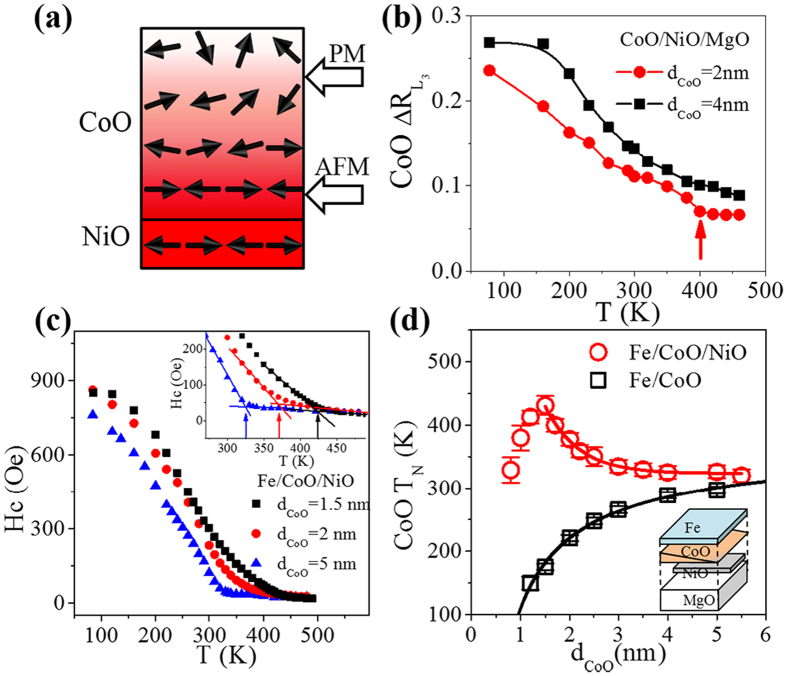
Study of AFM order propagation in CoO layer. (**a**) Schematic of AFM order in the AFM bilayer. (**b**) CoO *L*_*3*_ ratio difference 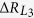 as a function of temperature for CoO/NiO (2 nm)/MgO(001) with a different CoO thickness. (**c**) Temperature dependence of Hc for Fe/CoO/NiO (2 nm)/MgO(001) with a different CoO thickness. Inset shows an enlargement of the Hc around T_N_. (**d**) T_N_ of CoO layer as a function of CoO thickness with and without a proximate 2-nm NiO layer. Inset in (**d**) shows the structure of the sample.

We have shown that exchange coupling in the CoO/NiO bilayer enhanced the CoO T_N_. This enhancement of the CoO ordering temperature was expected to be at a maximum near the CoO/NiO interface owing to the interfacial nature of exchange coupling. Thus, the ordering temperature of the CoO should gradually change along the thickness direction, as indicated by the schematic in Fig. 5(a). For a sufficiently thick CoO film, the ordering temperature of the outer part of the CoO layer is expected to be close to that of bulk CoO. However, this theory is hard to prove using XMLD measurements. Figure 4(b) shows the temperature-dependent 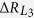 of 2 nm CoO in the CoO/NiO bilayer had a clear transition which indicated a T_N_ of ~400 K. In contrast, it only gradually decreased with temperature for 4 nm CoO, and no obvious transition could be distinguished. Thus, it was difficult to determine the ordering temperature of 4 nm CoO using XMLD measurements. As indicated in Fig. 5(a), different parts of the 4-nm CoO layer had different ordering temperature; thus, the XMLD measurements detected all the AFM information, and did not show the single phase transition.

To determine the CoO AFM order away from the AFM interface, we grew 2 nm Fe on top of CoO/NiO (2 nm)/MgO(001) with the CoO grown into a wedge shape, and used MOKE measurements to determine the ordering temperature of the CoO spins at the Fe/CoO interface through the interfacial exchange coupling. As shown in Fig. 5(c), the temperature-dependent Hc obtained from the magnetic hysteresis loops showed a clear transition. The T_N_ of 1.5 nm, 2 nm, and 5 nm CoO was determined to be 430 K, 380 K, and 330 K, respectively. Half of the sample was grown as Fe (2 nm)/CoO(wedge) without the NiO underlayer, as indicated by the inset in Fig. 5(d), thus both the Fe/CoO/NiO and Fe/CoO samples had identical growth conditions. Figure 5(d) shows the T_N_ at the Fe/CoO interface as a function of CoO thickness for both Fe/CoO/NiO and Fe/CoO samples. The T_N_ of CoO in the AFM bilayer was always higher than that without the NiO layer. Therefore, our results demonstrate that the exchange coupling at the CoO/NiO interface can significantly enhance the ordering temperature of CoO. Without the NiO underlayer, the T_N_ of the CoO layer followed the finite size scaling law well, as previously reported^13^. However, with the NiO layer, the T_N_ of the CoO first increased with thickness and then decreased exponentially when the CoO thickness was larger than 1.5 nm. In both Fe/CoO/NiO and Fe/CoO samples, the determined T_N_ for thick CoO saturated at ~300 K, which is close to the bulk T_N_ of CoO. The T_N_ measured for the CoO/NiO bilayer first increased with CoO thickness owing to the finite size effect. However, the induced AFM order of the CoO in CoO/NiO bilayer should not have a higher ordering temperature than the NiO layer, and the AFM order of the CoO spins in the outer layer weakens in thick CoO film, so the measured T_N_ decreased for CoO thicknesses above 1.5 nm. By fitting T_N_(d_CoO_) with an exponential decay function for d_CoO_ > 1.5 nm, we obtained a characteristic decaying thickness of ~0.7 nm. Our results indicate that the propagation length of AFM order in CoO is about 2–3 nm.

## Discussion

The anisotropy of CoO is about two orders of magnitude stronger than that of NiO^38^, thus the CoO spins in the CoO/NiO bilayer were always in-plane, whereas the spin orientation of the NiO layer was influenced by the competition between interfacial exchange coupling and lattice-strain-induced out-of-plane anisotropy^29^. The interfacial exchange coupling should be collinear coupling, because the thin NiO spins were driven to align along the film plane by the in-plane CoO spins. However, in the AFM bilayer with thick NiO, the AFM proximity effect weakened with distance from the CoO/NiO interface^24^,^25^,^26^, while the strain-induced anisotropy in the NiO persisted above 20 nm in our experiment. Thus, for NiO far from the CoO/NiO interface, the AFM NiO spin was aligned out of plane, while near the interface the AFM spin was in the canting state [Fig. 1(f)]. In this case, it is natural to suppose a vertical exchange spring spin alignment existed in the thicker NiO layers, as shown in Fig. 1(d), which has never been reported before. Within the detection length of the XMLD measurements, the NiO AFM spins were found to change from in-plane to the canting orientation with increasing NiO thickness at low temperature.

Figure 3(e,f) show that the NiO spins changed from in-plane (canting state) to out-of-plane with increasing temperature; this can be understood by the weakened exchange coupling strength at the NiO/CoO interface at high temperature. The AFM exchange coupling strength was also related to the AFM spin orientation, as described by 
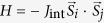
, where 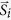 and 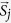 represent CoO and NiO spin, and 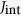 is the interfacial exchange coupling constant. Because the CoO spin was aligned in-plane, the AFM exchange coupling strength should have become smaller when the NiO spin rotated from in-plane to out-of-plane. It is therefore reasonable that a drop in CoO T_N_ was observed when the spin orientation of the proximate NiO layer changed from in-plane to out-of-plane. Monte Carlo simulation with some typical parameters qualitatively confirmed our experimental results (see Supplementary Fig. S2). The length of AFM order propagation in the CoO layer of the CoO/NiO bilayer was estimated to be 2–3 nm. In terms of previous reports on CoO/NiO superlattices, this value is similar to that in ref. 26 and smaller than that in ref. 25, which can be attributed to different film qualities resulting from the use of different growth methods. Moreover, such AFM order propagation in the AFM bilayer was also confirmed qualitatively by Monte Carlo simulation (see Supplementary Fig. S3).

In general, the XMLD signal should reflect the AFM spin alignment, so if the spin configuration in the NiO film is known, the NiO-thickness dependent XMLD signal in Fig. 2 can be quantitatively simulated. The NiO XMLD signal could be expressed as 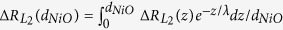
40, where 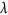 is the electron escape depth and 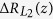 is the XMLD signal of the NiO layer at the position with depth 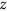 away from the CoO/NiO interface, and depends on the local spin canting angle 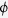 at 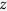, i.e. 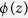, as defined in Fig. 1(d). 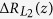
may be reasonably expressed as 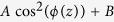
33. In Fig. 2(d), 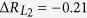 for in-plane aligned NiO spin, and 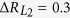 for out-of-plane aligned NiO spin at a higher temperature. Thus, we can determine B = 0.3 and A = −0.51. The 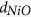-dependent spin profile was calculated by Monte Carlo simulation (see Supplementary part 2), and the 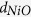-dependent 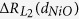 was then calculated by choosing a suitable value for 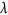. The green line in Fig. 2(d) shows the calculated 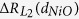 with 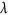 = 4.0 nm, which agrees well with the experimental result observed at low temperature. Our calculations further verified the model with the AFM exchange spring structure shown in Fig. 1(d). Based on the XMLD transition thickness in Fig. 2(d), the length scale of the AFM exchange spring was estimated to be ~6 nm at 78 K, and became smaller at higher temperature owing to the weakening of the exchange coupling.

It should be noted that the 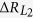 of thick NiO film depends on the vertical spin profile, which could be different in the systems with different NiO thickness. In our Monte Carlo simulations, the 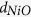-dependent spin profile was strongly dependent on the exchange coupling constants and AFM magnetic anisotropies, and these parameters are difficult to determine experimentally. XMLD measurements can only indicate the average AFM properties, and quantitatively determining the AFM spin profile across the film requires other techniques with layer specification. We note that the FM magnetization profile of magnetic heterostructures can be investigated by X-ray resonant magnetic reflectometry based on the X-ray magnetic circular dichroism effect^41^. Thus, the study of non-uniform AFM spin alignment in AFM films is likely to be possible using X-ray resonant magnetic reflectometry based on the XMLD effect.

In summary, we investigated the AFM proximity effect in a CoO/NiO/MgO(001) system on the basis of AFM spin orientation, Néel temperature, and AFM order propagation length. Owing to AFM interfacial exchange coupling, the NiO AFM spin underwent SRT from in-plane to out-of-plane with increasing NiO thickness, with the existence of a vertical exchange spring structure in thick NiO. The SRT had a great influence on the T_N_ of the CoO layer, and influenced the AFM order of the CoO within a limited depth of about 2–3 nm from the interface. The present work is expected to allow new designs for AFM-based devices that exploit the AFM proximity effect in AFM/AFM bilayers, where the AFM spin orientation and AFM ordering temperature can be manipulated without involving any FM layer.

## Methods

### Sample preparation

CoO/NiO/MgO(001) films were prepared by molecular beam epitaxy (MBE) in an ultra-high vacuum (UHV) chamber with a base pressure of 2 × 10^−10^ Torr. The single-crystal MgO(001) substrate was first annealed at 600 °C for 30 min in the UHV chamber, followed by the growth of a 10-nm MgO seed layer at 500 °C by e-beam evaporation. Subsequently, the NiO and CoO layers were prepared by reactive deposition of Ni and Co at an oxygen pressure of 1 × 10^−6^ Torr at room temperature (RT)^11^,^29^,^32^. A smooth surface was confirmed by the sharp reflection RHEED patterns. Film thickness was determined by the deposition rate (~1.0–2.0 Å/min), and was measured using a calibrated quartz thickness monitor. For thickness-dependence measurements, the CoO(NiO) film was grown in a wedge shape by moving the substrate behind a knife-edge shutter. To study the exchange coupling between CoO AFM spins and Fe FM spins, Fe film was grown on the bilayers by MBE at RT. *In situ* RHEED patterns indicated high quality epitaxial growth of both the fcc structured CoO (NiO) films and the bcc Fe film with an epitaxial relationship of Fe[100]//CoO[110]//NiO[110]//MgO[110] (see Supplementary Fig. S1). Finally, the sample was capped with a 3-nm MgO layer as a protective layer for MOKE measurements. For the samples subjected to XMLD measurements, a Al (4-nm)/MgO (1-nm) bilayer was capped on top of the CoO layer to prevent charging effects, and a 1-nm MgO layer was used to prevent a chemical reaction between the Al and CoO.

### XMLD measurements

XMLD measurements were performed at the bending-magnet Beamline 08B of the National Synchrotron Radiation Research Center (NSRRC) in Taiwan. The X-ray polarization was fixed along the horizontal direction and spectra were collected in total electron yield mode. The absorption spectra were collected for both normal (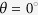) and grazing (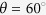 or 70°) X-ray incidence^29^,^31^,^32^. The linear background has been subtracted for all of the XAS spectra shown in this paper. Samples were mounted with the slope of the NiO wedge along the vertical direction so that the NiO thickness could be easily varied by moving the sample up and down to illuminate the sample at different positions. The X-ray beam size was estimated to be 100 μm along the vertical direction and 300 μm along the horizontal direction. Accordingly, the thickness variation in the vertical direction within the X-ray beam caused by the wedge shape was less than 0.2 nm. The sample temperature was adjustable in the range of 78–460 K with a precision of less than 0.1 K.

### MOKE measurements

The magnetic properties of the films were determined by MOKE measurement using a laser diode with a wavelength of 670 nm. By taking advantage of the small laser beam size (below 0.2 mm) of the MOKE apparatus, we were able to systematically perform thickness-dependent studies on the same wedge-shaped sample as that used in XMLD measurements. The sample temperature for the MOKE measurements was adjustable in the range of 80–500 K.

## Additional Information

**How to cite this article**: Li, Q. *et al.* Antiferromagnetic proximity effect in epitaxial CoO/NiO/MgO(001) systems. *Sci. Rep.* **6**, 22355; doi: 10.1038/srep22355 (2016).

## Supplementary Material

Supplementary Information
