# Supplementary material for: Antiferromagnetic proximity effect in epitaxial CoO/NiO/MgO(001) systems
Source: Sci Rep. 2016 Mar 2;6:22355. doi: 10.1038/srep22355 (PMC4773757; doi:10.1038/srep22355)
Supplement: Supplementary Information [file srep22355-s1.pdf]

Supplementary information

Antiferromagnetic proximity effect in epitaxial CoO/NiO/MgO(001) systems

Q. Li[1], J. H. Liang[1], Y. M. Luo[1], Z. Ding[1], T. Gu[1], Z. Hu[2], C. Y. Hua[2,3], H.-J. Lin[3], T. W. Pi[3], S. P. Kang[4], C. Won[4] and Y. Z. Wu[1*]

[1]Department of Physics, State Key Laboratory of Surface Physics and Collaborative Innovation Center of Advanced Microstructures, Fudan University, Shanghai 200433, People's Republic of China

[2]Max-Planck-Institut für Chemische Physik fester Stoffe, Nöthnitzer Str. 40, Dresden 01187, Germany

[3]National Synchrotron Radiation Research Center, Hsinchu 30076, Taiwan, Republic of China

[4]Department of Physics, Kyung Hee University, Seoul 130-701, Republic of Korea

1. RHEED characterization

The CoO/NiO/MgO(001) films were grown by molecular beam epitaxy (MBE). Fig. S1 shows the RHEED patterns for the substrate and each layer, and the sharp stripes in the RHEED patterns prove the high quality film growth and smooth surface of each layer.

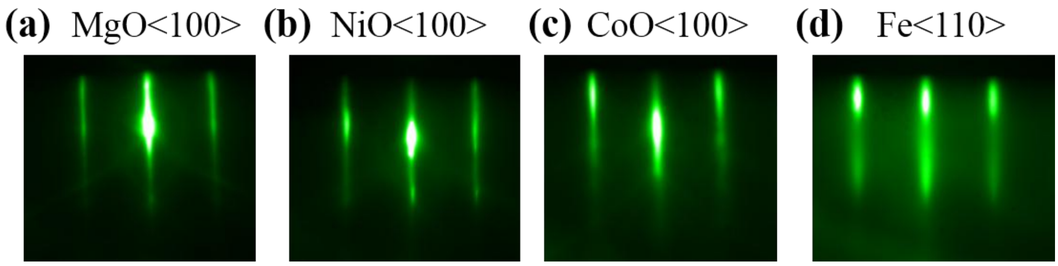

Figure S1│ RHEED patterns The typical RHEED patterns from (a) MgO(001) substrate (b) NiO/MgO(001) (c) CoO/NiO/MgO(001) and (d) Fe/CoO/NiO/MgO(001) with electron incidence direction along MgO<100>, NiO<100>, CoO<100> and Fe<110> respectively.

2. Monte Carlo simulation

In order to understand the physical origin of our experimental observation, we performed Monte Carlo simulation to study the spin profiles and the thickness-dependent ordering temperature in CoO/NiO bilayer. It is well known that the results of Monte Carlo simulation depend on the selected parameters, and our Monte Carlo simulation can qualitatively explain the thickness dependence of the ordering temperature due to the magnetic proximity effect at CoO/NiO interface.

We adopted the Heisenberg model for the free directional rotation of spin and simple cubic structure of Co^{2+} and Ni^{2+} with each lattice site interacting with six nearest-neighbor sites antiferromagnetically. This model is slightly different with the real spin arrangement in CoO and NiO crystal, but our simplified model is reasonable

enough to catch the physical origin and explain the observed experimental results. We used periodic boundary condition on the x-y dimension with 80x80 spin sites and free boundary condition in z dimension. The number of spin sites along z direction, interpreted as the thickness of a layer, can be varied in the simulation. The spin direction of the next iteration is determined by the effective field following Maxwell Boltzman statistics distribution. The effective field $\vec{H}_{i,\text{eff}}$ for a spin $\vec{S}_i$ is obtained from $\vec{H}_{i,\text{eff}} = -\delta E_i / \delta \vec{S}_i$, where E_i is the energy of spin $\vec{S}_i$. We did the initial 1000 iterations to stabilize the total energy of system, and then collected the data during the following 1000 iterations. The details about the simulation can be found in Ref. 1.

We only considered the exchange coupling energy and anisotropy energy terms in this AFM bilayer. Hence, the total energy of the system is given by:

$$\text{E}=\text{J}_1 \sum_{<i,i'>} \vec{S}_{1i} \cdot \vec{S}_{1i'} + \text{J}_2 \sum_{<i,i'>} \vec{S}_{2i} \cdot \vec{S}_{2i'} + \text{J}_{\text{int}} \sum_{<i,i'>} \vec{S}_{1i} \cdot \vec{S}_{2i'} - K_{z,1} \sum_i S_{z,i}^2 - K_{z,2} \sum_i S_{z,2i}^2$$

Where i and i' represent the lattice sites number, the normalized constants J_1, J_2, J_{int} are the exchange interaction energy constants and $\text{K}_{z,1}$ and $\text{K}_{z,2}$ are uniaxial anisotropy constants with easy axis along z direction in AF_1 and AF_2 layers, respectively, which corresponds to the uniaxial AFM anisotropy in AFM film. In the simulation, the AF_1 layer represents the CoO layer, and the AF_2 layer represents the NiO layer. In order to simulate the experimental phenomenon in CoO(AF_1)/NiO(AF_2) bilayers, the simulation parameters were reasonably chosen as $\text{J}_1 = -0.56$, $\text{J}_2 = -1$, $\text{J}_{\text{int}} = -0.78$, $\text{K}_{z,1} = -0.1$, $\text{K}_{z,2} = 0.0075$. Here, the exchange coupling energy strength J_2 of NiO was set as -1, and the value of J_1 for CoO was chosen as -0.56 based on the ratio of T_Ns between NiO and CoO. The interface exchange coupling strength J_{int}

was chosen as the average value of J_1 and J_2. Since the CoO AFM spins contain the in-plane anisotropy and the NiO spins have the out-of-plane anisotropy due to the film strain, we set $K_{z,1} < 0$ and $K_{z,2} > 0$ in the simulation.

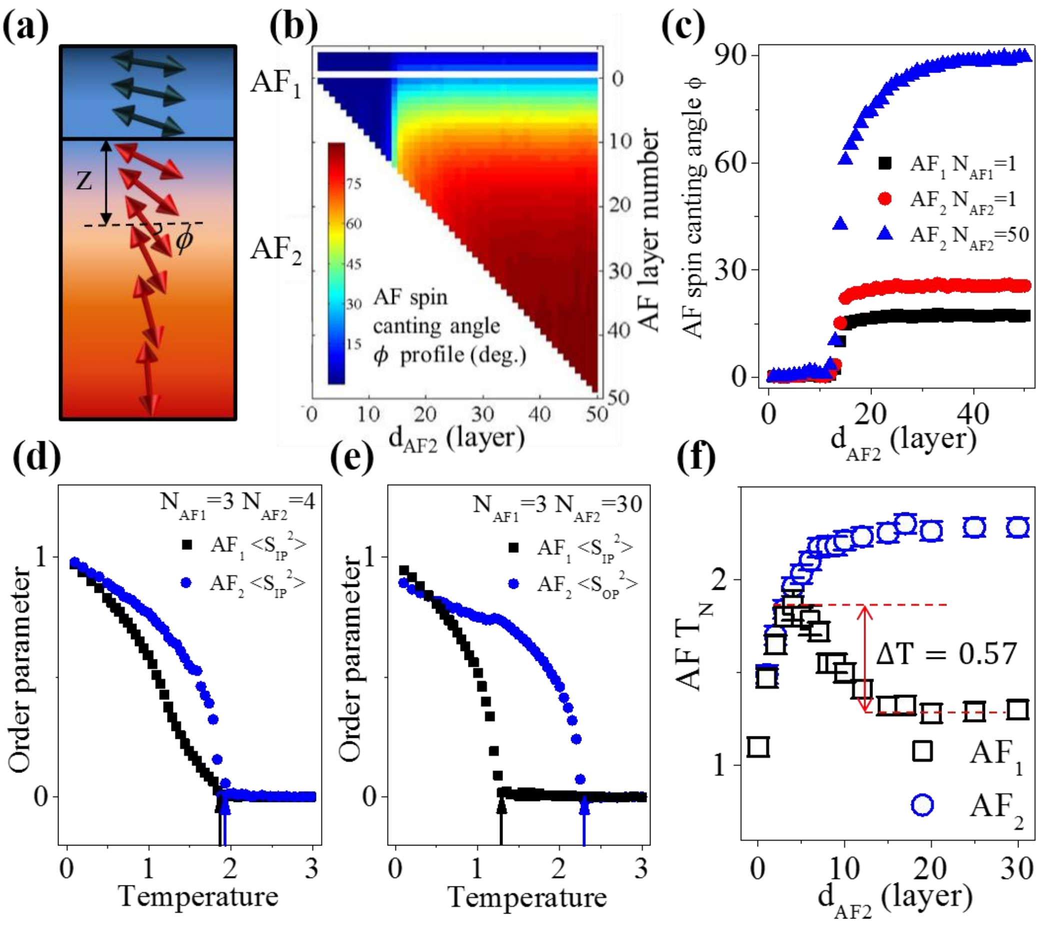

Figure S2│ Monte Carlo simulation of AFM SRT and spin-orientation-dependent T_N (a) Schematic drawing of spin configurations of AFM bilayers with thick AF_2 layer at low temperature. (b) Calculated AF spin canting angle ϕ of each AF layer as a function of AF_2 thickness at T=0.2. Inset is the color bar representing spin canting angle ϕ. (c) AF_2-thickness-dependent AF_1 and AF_2 spin canting angle ϕ at AFM interface (N_{AF1}=1, N_{AF2}=1) and farthest away from AFM interface (N_{AF2}=50). Temperature dependent AF_1 in-plane and AF_2 out-of-plane (in-plane) component of order parameter for (d) 3 AF_1 layers, 4 AF_2 layers and (e) 3 AF_1 layers, 30 AF_2 layers. (f) AFM T_N as a function of AF_2 thickness.

We first simulated the spin profile of AF_1 (3 layers)/AF_2 (30 layers) at lower temperature with T=0.2. As shown by the schematic drawings of AFM bilayer spin configuration in Fig. S2(a), the AF_1 spins align in the film plane, and the AF_2 spins gradually rotates from in-plane to out-of-plane, forming a vertical exchange spring structure. This spin structure is in consistent with our interpretation based on the XMLD measurement shown in Fig. 2 in the main text. Here we can calculate the spin canting angle ϕ of each AFM layer, which is defined as the angle between the AF spin orientation and the film surface. We calculated the ϕ distribution with different AF_2 layer thickness and fixed AF_1 layer thickness, as shown by the color map in Fig. S2(b). Fig. S2(c) also shows the spin canting angle ϕ as a function of d_{AF2} for the interface AF_1 layer, the interface AF_2 layer and the outermost AF_2 layer. It is very clear that a spin reorientation transition (SRT) happens at d_{AF2}~14 layers. For d_{AF2}<14 layers, all the spins in AF_1 layer and AF_2 layer align in the film plane with $\phi = 0^\circ$. For d_{AF2}>14 layer, the spin canting angle ϕ of the outermost AF_2 layer gradually approached to 90°, which means the out-of-plane spin alignment, however the spin canting angle of the interface AF_2 layer quickly increase at d_{AF2}~14 layers, then stabilizes at $\phi = 25.6^\circ$, so there is the vertical spin spiral across the AF_2 layer, as indicated by the color change in Fig. S2(b). It should be noted that the spin canting angle of AF_1 layer also has obvious change around the SRT thickness, but our XMLD measurement on CoO/NiO bilayer indicate that the CoO spins always align in the film plane. Such minor disagreement between the simulation and the experimental observation comes from the choice of the

simulation parameter, especially from the choice of the strength of $K_{z,1}$. In our simulation, the strength of $K_{z,1}$ is only one order stronger than that of $K_{z,2}$, but in real case the CoO anisotropy could be much stronger than the NiO anisotropy[2].

Based on the d_{AF2}-dependent spin profile in Monte Carlo simulation result, we calculated the d_{NiO}-dependent $\Delta R_{L_2}(d_{NiO})$ to compare with experiment result. Each layer contributes to the XMLD signal in the form of $\Delta R_{L_2}(\phi(z))e^{-z/\lambda}$[3], Where λ is the electron escape length, and $\Delta R_{L_2}(z)$ is the XMLD signal of the NiO layer at the position with depth z away from the CoO/NiO interface, and depends on the local spin canting angle ϕ at z. $\Delta R_{L_2}(\phi(z))$ may be reasonably expressed as $A\cos^2(\phi(z))+B$. In Fig. 2(d) in the main text, $\Delta R_{L_2}=-0.21$ for in-plane aligned NiO spin, and $\Delta R_{L_2}=0.3$ for out-of-plane aligned NiO spins at high temperature, thus we can determine B=0.3 and A=-0.51. By integrating all the AF_2 layers with the thickness of d_{AF2}, we can get the weighted averaged XMLD signal of d_{AF2} $\Delta R_{L_2}(d_{AF2})=\int_0^{d_{NiO}}(A\cos^2(\phi(z))+B)e^{-z/\lambda}dz/d_{AF2}$. Then by choosing a suitable value of λ, the d_{AF2}-dependent $\Delta R_{L_2}(d_{AF2})$ was calculated. Supposing 1 layer AF_2 in the simulation represents 0.4 nm NiO in the experiment, the calculated curve of $\Delta R_{L_2}(d_{AF2})$ with λ =4.0 nm best agrees with the experiment result at low temperature, shown as the green line in Fig. 2(d) in the main text.

The Néel temperature (T_N) could be obtained through Monte Carlo simulation. Order parameter of each lattice site was defined as $O_{i,j,k}=(-1)^{i+j+k}\vec{S}_{i,j,k}$ with i, j, k as the lattice cite number along x y and z dimension respectively. Here in order to study the in-plane and out-of-plane component of order parameter separately, we define the

in-plane component as $S_{IP} = \sqrt{[(\sum_i^N O_i)_x^2 + (\sum_i^N O_i)_y^2] / N}$ and the out-of-plane component as $S_{OP} = \sqrt{(\sum_i^N O_i)_z^2 / N}$, with N as the total number of the lattice sites. Temperature-dependent order parameter in Fig. S2(d) show the AF_1 and AF_2 layer have similar T_N in AF_1 (3 layers)/AF_2 (4 layers). However in AF_1 (3 layers)/AF_2 (30 layers) bilayer [Fig. S2(e)], the T_N of AF_2 is much lower than that of AF_1. Fig. S2(f) shows the systematic simulation of T_N as a function of AF_2 thickness, where the T_N of AF_2 follows the finite size scaling behavior while T_N of AF_1 first increases for thin AF_2 layer and then decreases about 30% for thicker AF_2 layers. As shown in Fig. S2(b) and (c), AF_2 spins rotate from in plane to out of plane at the critical AF_2 thickness of 14 layers at T=0.2. Moreover, we found this critical SRT thickness decreases with increasing temperature, and reaches about 5 layers for T=1.8. Therefore, the drop of AF_1's T_N could be attributed to the spin reorientation of the adjacent AF_2 layer.

We also simulated the distribution of the ordering temperature in a thick AF_1 layer. The simulation was performed in a AF_1(15 layers)/AF_2(5 layers) bilayer system. Here, the AF_2 layer thickness was chosen as 5 layer to get the in-plane spin alignment. The T_N of the AF_2 layer was determined as ~2.3. Figure S3(a) shows the temperature dependent order parameter S_{IP} of each AF_1 layer, which clearly indicates the different ordering temperature for different layer. The T_N of each AF_1 layer was plot in Fig. S3(b), which decreases quickly while the AF_1 layer is away from the interface, and reaches T_N of bulk AF_1 for the 7th layer. This simulated results indicated that the AFM order in AF_1

layer induced by the magnetic proximity effect can only propagates up to 7 layers. We also simulated the T_N in the $AF_1/AF_2(5)$ bilayer system with different AF_1 thickness, and found that the ordering temperature of outmost AF_1 layer decreases with the AF_1 thickness, and reach a constant value for d_{AF1}>7 layers.

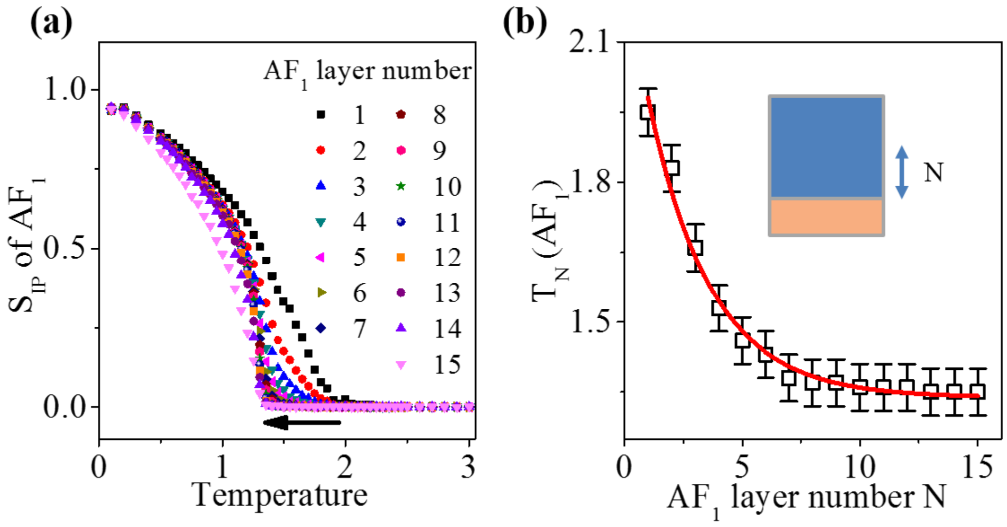

Figure S3│ Monte Carlo simulations of distribution of AF_1 T_N in AFM bilayers (a) The simulated temperature-dependent order parameter S_{IP} of each AF_1 layer in AF_1(15 layers)/AF_2(5 layers) bilayer system. (b) The T_N of each AF_1 layer derived from the data in (a). The inset in (b) show the sample structure.

In conclusion, the Monte Carlo simulation results well support our experimental observation in CoO/NiO bilayer system reported in the main text. The competition between the strain-induced magnetic anisotropy and interfacial exchange coupling leads to the SRT of soft NiO layer. The AFM ordering temperature in CoO layer was greatly enhanced by interface exchange coupling at interface of AFM bilayer, and such enhancement of the CoO T_N is related to the spin orientation in NiO layer. Moreover,

the extra AFM order in CoO layer induced by the interfacial exchange coupling at NiO/CoO interface only locates within a limited thickness range.

Reference:

1 Seok, J. H., Kwon, H. Y., Hong, S. S., Wu, Y. Z., Qiu, Z. Q. & Won, C., In-plane spin reorientation transition in a two-dimensional ferromagnetic/antiferromagnetic system studied using Monte Carlo simulations, *Phys. Rev. B*, **80**, 174407 (2009).

2 Schrön, A. *et al.* Crystalline and magnetic anisotropy of the 3d-transition metal monoxides MnO, FeO, CoO, and NiO, *Phys. Rev. B* **86**, 115134 (2014).

3 Nakajima, R., Stöhr, J. & Idzerda, Y. U. Electron-yield saturation effects in L-edge x-ray magnetic circular dichroism spectra of Fe, Co, and Ni, *Phys. Rev. B* **59**, 6421 (1999).
